# Social context matters: the role of social support and social norms in healthcare solidarity

**DOI:** 10.1093/eurpub/ckac129.164

**Published:** 2022-10-25

**Authors:** MA Meijer, AEM Brabers, JD De Jong

**Affiliations:** 1 Healthcare System and Governance, NIVEL, Utrecht, Netherlands; 2 Faculty of Health, Medicine and Life Sciences, Maastricht University, Maastricht, Netherlands

## Abstract

**Background:**

In many European countries, including the Netherlands, the healthcare system is based upon solidarity. It is important that public support for solidarity-based systems is sufficient, to ensure that people remain willing to contribute to them. Although support is generally high, as indicated by high levels of willingness to pay for the healthcare costs of others, there are differences between groups. Previous research has focused on individual and institutional characteristics when explaining these differences. However, people's social context may also play a role. Little research has been conducted into this. To fill this gap, we examined the role of perceived social support and social norms in order to explain differences in the willingness to pay for other people's healthcare costs.

**Methods:**

A questionnaire was sent to a representative sample of 1,500 members of the Dutch Healthcare Consumer Panel in November 2021 (56% response rate, N = 837). The relationship between the social context of people and their willingness to pay was studied via logistic regression analysis.

**Results:**

Higher levels of perceived social support are associated with higher levels of willingness to pay for other people's healthcare costs (p = 0.038). We also found that willingness to pay is higher when someone's social context is more supportive of the solidarity-based healthcare system (p = 0.001). Contrary to our expectations, the effect of social norms does not differ between people who perceive low and high levels of socials support.

**Conclusions:**

The degree to which people feel connected to others and the degree to which someone's social context supports the solidarity-based healthcare system affect the willingness to contribute to the healthcare system. Our results suggest that the social context of people has to be taken into account in both policy and research that addresses healthcare solidarity, next to individual and institutional characteristics.

**Key messages:**

• Social support and social norms play a role in the willingness to pay for healthcare costs of others.

• People's social context must be taken into account in policy and research on healthcare solidarity.

